# Feeding Ecology in Oligocene Mylodontoid Sloths (Mammalia, Xenarthra) as Revealed by Orthodentine Microwear Analysis

**DOI:** 10.1007/s10914-017-9405-x

**Published:** 2017-07-28

**Authors:** Daniela C. Kalthoff, Jeremy L. Green

**Affiliations:** 1 Department of Zoology, Swedish Museum of Natural History, Box 50007, SE–104 05 Stockholm, Sweden; 2 Department of Geology, Kent State University at Tuscarawas, 330 University Drive NE, New Philadelphia, OH 44663 USA

**Keywords:** Dental microwear, Scanning electron microscopy, Stereomicroscopy, Folivora, Paleodiet, Orthodentine

## Abstract

Recently, dental microwear analysis has been successfully employed to xenarthran teeth. Here, we present new data on use wear features on 16 molariforms of *Orophodon hapaloides* and *Octodontotherium grande*. These taxa count among the earliest sloths and are known from the Deseadan SALMA (late Oligocene). Modern phylogenetic analyses classify *Octodontotherium* and *Orophodon* within Mylodontoidea with whom they share lobate cheek teeth with an outer layer of cementum and a thick layer of orthodentine. Similar target areas of 100μm^2^ were analyzed on the orthodentine surface of each tooth by stereomicroscopic microwear and by SEM microwear. Results were unlike those of extant sloths (stereomicroscopic microwear: *Bradypus*, *Choloepus*) and published data from fossil sloths (SEM microwear: *Acratocnus*, *Megalonyx*, *Megatherium*, *Thinobadistes*); thus, both approaches independently indicate a different feeding ecology for the Oligocene taxa. The unique microwear results suggest that both taxa fed on plant material with low to moderate intrinsic toughness (foliage, twigs) but also proposes intake of tougher food items (e.g., seeds). Frequent gouging of the tooth surfaces can be explained by exogenous influence on microwear, such as possible intake of abrasive grit. We suggest an unspecialized herbivorous diet for *Octodontotherium* and *Orophodon* utilizing diverse food resources of their habitat. These interpretations support the reconstruction of (1) Deseadan environments as open habitats with spreading savannas/grasslands and (2) both taxa as wide-muzzled bulk feeders at ground level.

**Electronic supplementary material:**

The online version of this article (doi:10.1007/s10914-017-9405-x) contains supplementary material, which is available to authorized users.

## Introduction

*Orophodon* and *Octodontotherium* are among the earliest definite sloths and are known from the Deseadan South American Land Mammal Age (late Oligocene) (Gaudin and Croft 2015). As Orophodontidae, they have long been considered an early offshoot in sloth phylogeny (Hoffstetter 1956). However, modern analyses classify *Octodontotherium* and *Orophodon* within Mylodontoidea with whom they share lobate cheek teeth with an outer layer of cementum and a thick layer of orthodentine, yet are different in showing an only narrow vasodentine center (Gaudin 2004; Carlini and Scillato-Yané 2004; Pujos and De Iuliis 2007; Kalthoff 2011). Ameghino (1895) described two distinct species from Patagonia, the large-sized *Octodontotherium grande* and the smaller *Orophodon hapaloides*. Remains of these Oligocene sloth taxa mainly consist of a number of teeth and some few postcranial elements (Hoffstetter 1956) plus a well-preserved but edentulous skull from *O. grande* (Patterson et al. 1992; Shockey and Anaya 2011: fig. 2b). Vizcaíno et al. (2012) indicated a body mass of 700 kg for *Octodontotherium grande* (on the basis of a *Glossotherium* skull), while Shockey and Anaya (2011) estimated a body mass half as much (based on the only *Octodontotherium* skull preserved). On the basis of the dimensions of the lower lobate teeth of *Octodontotherium* and *Orophodon*, respectively (Hoffstetter 1956); tooth size of the latter is about 30% smaller than the first. Even so, our knowledge of the paleobiology and ecology of these early mylodontids is very limited (but see Shockey and Anaya 2011).

### Microwear

Microwear analysis is an established method for evaluating use wear features on the occlusal tooth surfaces of both extant and fossil vertebrates as direct proxies for dietary behavior. These wear features are influenced by the properties of ingested food items (e.g., grass, leaves, fruits, insects, meat) or exogenous items (e.g., dust, grit), and have a rapid turnover of several days to one or two weeks (Teaford and Oyen 1989; Hoffmann et al. 2015). Several microscopic techniques have been used for microwear analysis: scanning electron microscopy (e.g., Rensberger 1978; Walker et al. 1978), stereomicroscopy (e.g., Solounias and Semprebon 2002; Semprebon et al. 2004, 2016; Merceron et al. 2005; Koenigswald et al. 2010; Rivals et al. 2010), and confocal microscopy (e.g., Scott et al. 2005, 2006; Ungar et al. 2008; Schulz et al. 2010; Calandra and Merceron 2016).

Right from the beginning of microwear analysis, tooth enamel has been the target tissue. However, recently, (ortho-)dentine has come into focus in taxonomic groups lacking enamel, such as xenarthrans (e.g., Oliveira 2001; Green 2009a, 2009b; Green and Resar 2012; Haupt et al. 2013; Resar et al. 2013). As reviewed by Green and Kalthoff (2015), microwear studies have proven feasible with all three microscopic techniques and offer insight into feeding habits of xenarthrans.

Here, we present new data on use wear features on 16 upper and lower molariform teeth (MF, mf) of *Orophodon hapaloides* (*n* = 5), *Octodontotherium grande* (*n* = 9), and *Orophodon* vel *Octodontotherium* (*n* = 2) representing 16 individuals in total. The same casts were examined at similar target areas using stereomicroscopy (DCK) and scanning electron microscopy (JLG). Both techniques have the ability to discern species-specific differences in sloth microwear (stereomicroscopy: Green 2009a, 2009b; SEM: Green and Resar 2012; Resar et al. 2013; Green and Kalthoff 2015; McAfee and Green 2015). Usually, only one methodology (either stereomicroscopy or SEM) is applied at a time to study microwear. However, more robust and objective interpretations of microwear are likely when different methods are used to study the same teeth. Stereoscopic and SEM analyses are inherently different methods in terms of illumination, magnification, surface area analyzed, and variables quantified, so we did not statistically compare light microscopic and SEM results with one another. Rather, we independently interpret our results from each method based on direct comparison with other sloths analyzed using the same respective technique.

### Motivation of the Study

Based on muzzle anatomy, extinct giant sloths have been characterized as bulk feeders (broad muzzle) or selective feeders (narrow muzzle) (Bargo et al. 2006). Examining the skull morphology of *Octodontotherium grande*, Shockey and Anaya (2011) suggested these animals as – possibly grazing – bulk feeders at ground level in predominately dry, savanna-like habitats. We test this assumption by analyzing microwear data in the same samples using two independent approaches (stereomicroscopy and SEM) to provide the most detailed, robust results.

### Hypothesis

The following hypotheses guided our microwear analysis in the Oligocene species *Orophodon hapaloides* and *Octodontotherium grande*:

1. Microwear features are different from browsers such as the extant sloths *Bradypus variegatus* and *Choloepus didactylus*.
2. Microwear features are similar to those of one or more taxa of stratigraphically younger, previously sampled ground sloths.

## Material and Methods

### Material

We analyzed 16 upper (MF) and lower (mf) molariforms of *Orophodon hapaloides* (2 MF, 3 mf), *Octodontotherium grande* (5 MF, 3 mf, 1 MF/mf), and *Orophodon* vel *Octodontotherium* (2 mf) representing 16 individuals in total. All molariforms but those of MNHN DES 250 (left MF 3–5, *O. grande*) and MNHN DES 277 (left mf 2–4, *O. hapaloides*) were isolated; of MNHN DES 250 and 277 we included data from only one molariform each in our calculation of mean species values and the resulting statistical comparison among taxa. Some specimens are figured in Hoffstetter (1956): figs. 1, 2, and 5; 1958: fig. 42) and Pujos and De Iuliis (2007): fig. 2a-d). Because of the rareness of available and suitable teeth for dental microwear, we could not follow the standard protocol in analyzing only one tooth position or two corresponding teeth in upper and lower jaws. The tooth sample presented in this study is therefore random.

**Fig. 1:**
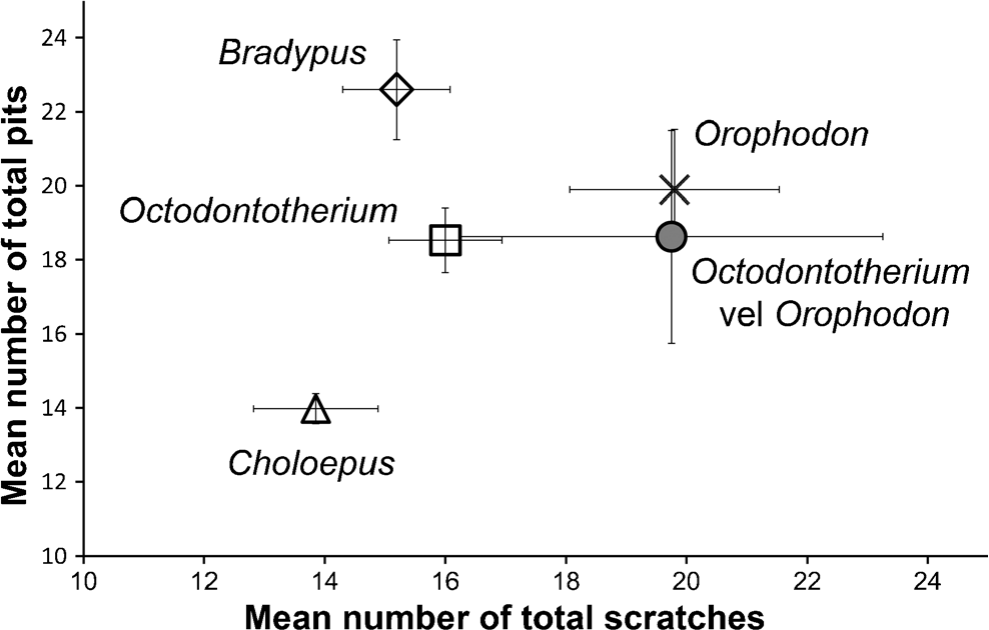
Bivariate plot comparing mean values of total number of fine and coarse scratches (TS) versus total number of small and large pits (TP) for all taxa analyzed by stereomicroscopic microwear

**Fig. 2:**
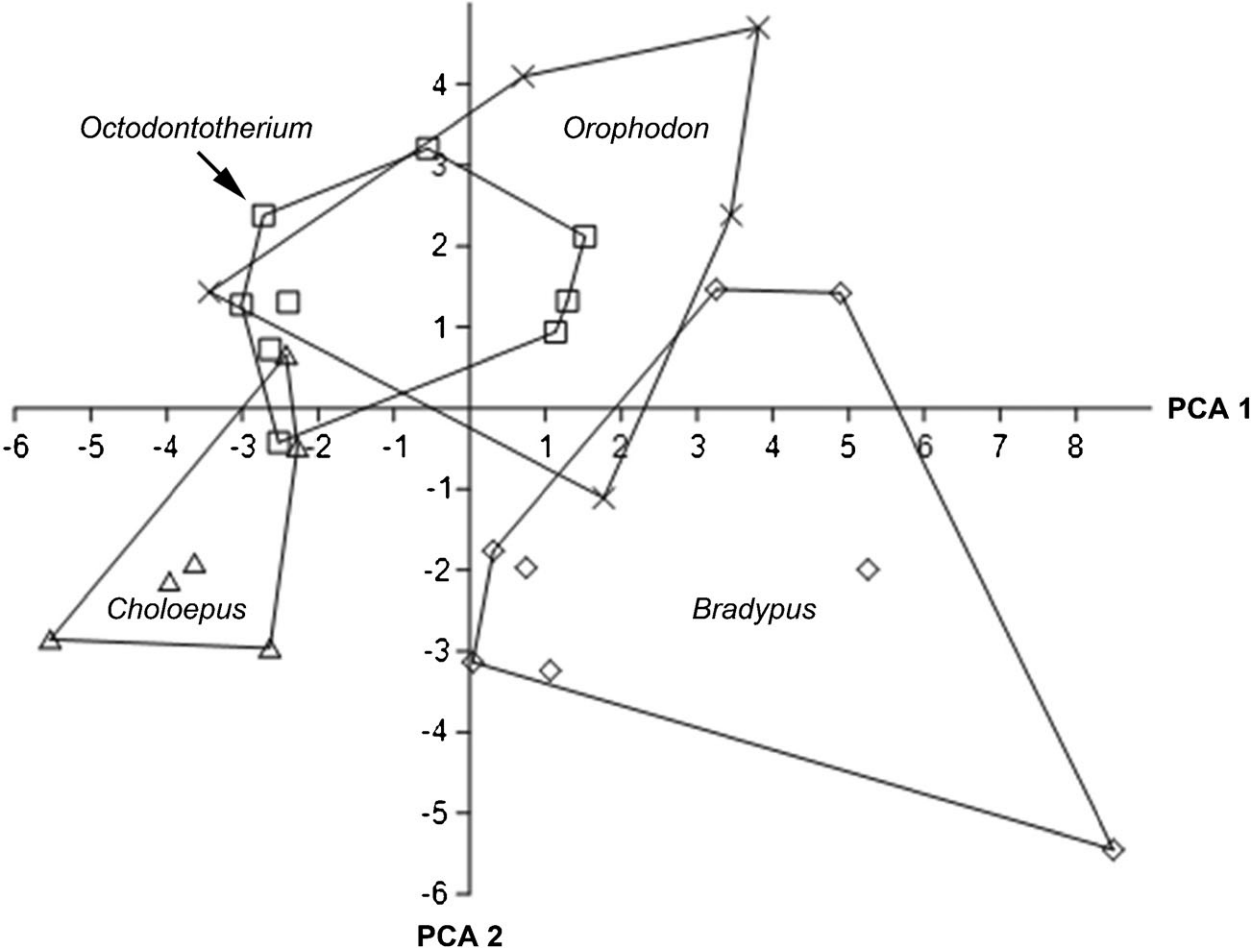
PCA on stereomicroscopic microwear variables: number of small pits; number of large pits; number of coarse scratches; presence/absence of puncture pits. Convex hulls embrace the areas taken by the each taxon

The fossil material was collected by André Tournouër at the Patagonian locality La Flecha (Deseadan SALMA, late Oligocene) and is housed at the Musée National d’Histoire Naturelle in Paris, France (MNHN). Comparative material from extant sloths (*Bradypus variegatus*, *n* = 8; *Choloepus didactylus*, *n* = 6) for the stereoscopic analysis comes from the Swedish Museum of Natural History in Stockholm, Sweden (NRM). The list of the specimens studied for the first time is given in Tables 1 and 6 (fossil taxa) and Table 2 (extant taxa).

**Table 1:** Mean stereoscopic microwear variables for all fossil individuals, with species mean and standard deviation

Specimen	Tooth	SP	LP	TP	FS	CS	TS	G	PP
*Octodontotherium grande*									
MNHN-DES 236	Rmf4	9.25	5.25	14.5	10.25	8.75	19	1	1
MNHN-DES 237	LMF4	13.5	4.5	18	7	6.25	13.25	1	1
MNHN-DES 238	RMF4	12.75	8.25	21	11.5	9.75	21.25	0	1
MNHN-DES 239	Lmf3	15	6	21	8.25	7.25	15.5	1	1
MNHN-DES 245	LMF5	12	8.5	20.5	7.75	8.75	16.5	1	1
MNHN-DES 246	Lmf4	10.5	6	16.5	7.25	6	13.25	1	1
MNHN-DES 250	LMF4	13.5	8.5	22	9.75	7.75	17.5	1	1
MNHN-DES 251	MF/mf?	11.5	5.25	16.75	6.25	7.25	13.5	1	1
MNHN-DES 252	RMF4	11.75	4.75	16.5	7.75	6.5	14.25	1	1
Mean		**12.19**	**6.33**	**18.53**	**8.42**	**7.58**	**16.00**	**0.89**	**1.00**
St Dev		**1.73**	**1.64**	**2.65**	**1.72**	**1.28**	**2.82**	**0.33**	**0.00**
*Orophodon hapaloides*									
MNHN-DES 260	mf1/mf2	15.5	9	24.5	12.25	11.75	24	1	1
MNHN-DES 267	Rmf4	12.25	6.5	18.75	11	12.25	23.25	1	1
MNHN-DES 268	RMF2	10.5	4.25	14.75	7	7.75	14.75	0	1
MNHN-DES 269	LMF3/MF4	9.5	10	19.5	8.25	9.25	17.5	0	1
MNHN-DES 277	Lmf4	12.25	9.75	22	7.5	12	19.5	1	1
Mean		**12.00**	**7.90**	**19.90**	**9.20**	**10.60**	**19.80**	**0.60**	**1.00**
St Dev		**2.28**	**2.47**	**3.66**	**2.30**	**2.00**	**3.89**	**0.55**	**0.00**
*Octodontotherium* vel *Orophodon*									
MNHN-DES 233	mf2?	11.25	4.5	15.75	7.75	5	12.75	1	1
MNHN-DES 234	mf2?	14	7.5	21.5	9.75	10	19.75	1	0
Mean		**12.63**	**6.00**	**18.63**	**8.75**	**7.50**	**16.25**	**1.00**	**0.50**
St Dev		**1.94**	**2.12**	**4.07**	**1.41**	**3.54**	**4.95**	**0.00**	**0.71**

The values bolder here reflect mean and standard variation

Molariforms from the maxilla are denoted by MF, those from the mandible by mf; L = left, R = right

Abbreviations: *SP* mean number of small pits, *LP* mean number of large pits, *TP* mean number of total pits, *FS* mean number of fine scratches, *CS* mean number of coarse scratches, *TS* mean number of total scratches, *G* gouges, *PP* puncture pits, 0 = feature absent, 1 = feature present

### Methods

All teeth were cleaned, molded, and cast applying the protocol of Solounias and Semprebon (2002). All casts were directly compared with the original occlusal surfaces using a stereomicroscope to exclude loss or alteration of features during preparation. Taphonomic alteration of fossil specimens was accounted for by directly comparing non-occlusal and occlusal surfaces in each tooth. Teeth showing similar microwear patterns on both surfaces were rejected due to the likelihood of alteration of the microwear signature (Teaford 1988). In addition, if microwear was absent on the chewing surface of a tooth, that specimen was also considered altered and rejected, as ante-mortem microwear was likely obliterated by post-mortem processes (King et al. 1999).

### Stereomicroscopic Microwear

Representative target areas (four for fossil sloths and two for extant sloths) of 100 μm^2^ were examined on the orthodentine surface of each tooth under incident light with a Zeiss Discovery V12 stereomicroscope at 70 x magnification using an ocular reticle (following Solounias and Semprebon 2002). Four quantitative (number of small pits = SP, number of large pits = LP, number of fine scratches = FS, number of coarse scratches = CS [including hypercoarse scratches]) and two qualitative parameters (presence/absence of gouges = G and puncture pits = PP) were examined by only one observer (DCK). In addition, the total number of pits (TP) and the total number of scratches (TS) were calculated. Raw and averaged counting results were compared to new data from two extant sloths: the Brown-throated sloth *Bradypus variegatus*, a selective browsing, obligate folivore and Linnaeus’s Two-toed sloth *Choloepus didactylus*, a browser with a more varied herbivorous diet.

### SEM Microwear

To make data from Oligocene sloths directly comparable to previously collected SEM data from extant and extinct sloths, we followed the methods of Green and Resar (2012), Resar et al. (2013), and Green and Kalthoff (2015). Casts were mounted on 25.4 mm aluminum stubs using standard carbon adhesive tables (Electron Microscopy Sciences, Inc.), with a layer of colloidal silver liquid (Electron Microscopy Sciences, Inc.) applied to the base of the stub to improve electron dispersal and adhesion of the cast to the stub. Mounted casts were sputter coated with a thin layer (10 nm) of platinum using an Electron Microscopy Sciences, Inc. 150 T S sputter coater and imaged using a JEOL JSM-6010PLUS/LV scanning electron microscope located at Kent State University at Stark. The wear surface imaged was always oriented normal to the electron beam in the specimen chamber. Two non-overlapping images were captured at 500× (with an operating voltage of 20 kV using secondary electrons) along the orthodentine layer on the mesial wear facet of each molariform. For DES 250, images were captured for M2, M3, and M4 and included in the original image file, totaling 36 images. Each image was resized to 1000 × 800 pixels and the brightness/contrast manually adjusted such that the lightest pixel was white and the darkest pixel black. A 100 μm × 100 μm square was digitally constructed and centered over the area of highest density of visible microwear features in each image. All digital adjustments were made in ImageJ (U.S. National Institute of Health, Bethesda, Maryland; http://imagej.nih.gov/ij). To reduce bias inherent in microwear counting, all images were randomized with specimen and taxonomic information removed (creating a blind analysis). Additionally, ten random images were duplicated and placed within the randomized image file to permit measurement of observer consistency in counting. All images were analyzed by only one of us (JLG) using the semi-automated custom sofware package, Microware 4.02 (Ungar 2002). The major and minor axis endpoints of all features within the 100 μm × 100 μm square were marked, and a length/width ratio of 4:1 was maintained to automatically discriminate scratches from pits. We focused on four variables recorded by the program: 1) number of scratches (S), 2) number of pits (P), 3) feature minor axis length, i.e., feature width in μm (FW), and 4) degree of parallelism in feature orientation (R), from 0 to 1, with 0 meaning features had a completely random orientation and 1 representing features with perfectly parallel alignment. These same four variables were analyzed by Green and Resar (2012), Resar et al. (2013), and Green and Kalthoff (2015).

### Statistics

Statistical tests for stereoscopic microwear were performed using PAST v.3.14 (Hammer et al. 2001). The mean number of small, large, and total pits and fine, coarse, and total scratches were calculated in each species and bivariate plots were constructed. We also calculated percentages of individuals in each taxon showing gouges and/or puncture pits. Normality of all variables was assessed using Shaprio-Wilk tests, with Levene tests used to test homogeneity of variance.

A one-way analysis of variance (ANOVA) with Tukey’s comparisons of species pairs was run for each numerical variable with normal distribution to test for significant differences between species. A Kruskal-Wallis test for equal medians was used for the two non-numerical (presence/absence) variables to identify significant differences between species.

We ran a principal component analysis (PCA) on the correlation matrix with four independent variables showing significant differences between taxa (number of small pits, number of large pits, number of coarse scratches, and percentage presence of puncture pits). The cut-off for the loadings is +/− 0.7. This allowed us to isolate the dominant use-wear features for each taxon.

For SEM microwear, statistical testing was done in SPSS (Statistical Package for Social Sciences, Inc.) v.22. Intraobserver differences for the ten duplicated images from 36 total image file were analyzed using a Wilcoxon Signed Rank Test (four total tests, one per variable). Although data from LMF2 and LMF3 of DES 250 were included in our test of intraobserver variation (see Supplementary Table 3), we only included variables from the LMF4 of this specimen in our calculation of mean variables for *Octodontotherium* and in our statistical analysis between *Octodontotherium* and other sloths. The LMF4 was chosen because it represents the most common tooth category in our random sample of *Octodontotherium* teeth, thereby reducing potential intra-tooth variation in microwear. We statistically compared new data from Oligocene sloths with comparable data (collected using the same methodology and the same observer (JLG)) from previously sampled extant and fossil sloths (Green and Resar 2012; Resar et al. 2013; Green and Kalthoff 2015). Normality of all four variables was assessed using Shaprio-Wilk tests, with Levene tests used to test homogeneity of variance. For normally distributed variables with equal variance, we ran a One-way ANOVA, followed by Tukey’s post-hoc tests for all pairwise combinations. For non-normal variables, we used Kruskal-Wallis tests, followed by Dunn-Bonferroni post-hoc tests for all pairwise combinations. We also conducted a hierarchical cluster analysis among all mean variables per species (using nearest neighbor, with taxon as the grouping variable, and all variables transformed to Z-scores to standardize data) to get an overall measure of similarity in microwear signature between Oligocene sloths and previously sampled sloths.

Because of small sample size, the two “*Orophodon* vel *Octodontotherium*” specimens were excluded from statistical testing but are still reported in the summary statistics and on the bivariate graphs.

All data generated or analyzed during this study are included in this published article and Supplementary Tables 1, 2, and 3.

**Table 2:** Mean stereoscopic microwear variables for all extant individuals, with species mean and standard deviation

Specimen	Tooth	SP	LP	TP	FS	CS	TS	G	PP
*Bradypus variegatus*									
NRM 580502	LMF4	10.5	18	28.5	10	7.5	17.5	1	0
NRM 580538	LMF4	14.5	11.5	26	10	9.5	19.5	1	0
NRM 581032	LMF4	9.5	10.5	20	5.5	6	11.5	1	1
NRM 581211	LMF4	9.5	9	18.5	6.5	7.5	14	1	0
NRM 581503	LMF4	13	10	23	5.5	10	15.5	0	0
NRM 581552	RMF4	10	9.5	19.5	8	5	13	0	0
NRM 581557	LMF4	10	9.5	19.5	8.5	7	15.5	1	0
NRM 581564	LMF4	12.5	13.5	26	7.5	7.5	15	1	0
Mean		**11.19**	**11.44**	**22.63**	**7.69**	**7.50**	**15.19**	**0.75**	**0.13**
St Dev		**1.89**	**3.02**	**3.80**	**1.79**	**1.65**	**2.51**	**0.46**	**0.35**
*Choloepus didactylus*									
NRM 580717	RMF3	7.5	7	14.5	6.5	6	12.5	1	0
NRM 581540	LMF3	10	5.5	15.5	6	8	14	1	1
NRM 586554	LMF3	7.7	5.7	13.4	6.3	6.3	12.6	1	1
NRM 593602	LMF3	7	6	13	7	10	17	1	0
NRM 593606	RMF3	9	5.5	14.5	12	4.5	16.5	0	0
NRM 601111	RMF2	8.5	4.5	13	7.5	3	10.5	1	0
Mean		**8.28**	**5.70**	**13.98**	**7.55**	**6.30**	**13.85**	**0.83**	**0.33**
St Dev		**1.11**	**0.81**	**1.01**	**2.24**	**2.48**	**2.51**	**0.41**	**0.52**

The values bolder here reflect mean and standard variation

Molariforms from the maxilla are denoted by MF; L = left, R = right

Abbreviations: *SP* mean number of small pits, *LP* mean number of large pits, *TP* mean number of total pits, *FS* mean number of fine scratches, *CS* mean number of coarse scratches, *TS* mean number of total scratches, *G* gouges, *PP* puncture pits, 0 = feature absent, 1 = feature present

**Table 3:** Pairwise comparisons between the analyzed living and fossil sloth species depicting significant differences (*p* < 0.05) of SP (number of small pits), LP (number of large pits), TP (total number of small and large pits), CS (number of coarse scratches), TS (total number of fine and coarse scratches), and PP (presence of puncture pits)

	*Orophodon hapaloides*	*Bradypus variegatus*	*Choloepus didactylus*
*Octodontotherium grande*	CS	LP, PP	SP, TP, PP
*Orophodon hapaloides*		LP, CS, TS, PP	SP, TP, CS, TS, PP
*Bradypus variegatus*			SP, LP, TP

Fine scratches and presence of gouges were not significantly different between species

## Results

### Stereomicroscopic Microwear

The microwear results are summarized in Tables 1, 2, 3 , 4, and 5; raw counts are included in Supplementary Tables 1 and 2. One-way ANOVA results show that the number of coarse scratches is the only parameter being significantly different between *Octodontotherium* and *Orophodon* with the latter showing higher values. In both taxa, hypercoarse scratches are present. *Octodontotherium* differs from *Bradypus* in lower values of mean number of large pits and in a 100% presence of puncture pits. *Octodontotherium* significantly differs from *Choloepus* in having higher values of number of small pits and total pits and in a 100% presence of puncture pits. *Orophodon* is significantly different from *Bradypus* in having fewer large pits but higher numbers of coarse scratches and total scratches plus showing a 100% presence of puncture pits. *Orophodon* differs significantly from *Choloepus* in showing higher values for number of small pits, number of total pits, number of coarse scratches, number of total scratches, and having a 100% presence of puncture pits. The two extant species *Bradypus* and *Choloepus* differ regarding all three parameters of pits, with *Bradypus* showing significantly higher values. Individual data points for *Bradypus* show no overlap with those for *Choloepus* for the number of pits of any kind: *Bradypus* has minimum values of 18.5 while *Choloepus* has maximum values of 15.5 for this parameter. Fine scratches and presence of gouges were not significantly different between any of the species (Tables 3 and 4).

**Table 4:** ANOVA test results for the numerical parameters of stereoscopic microwear (small pits, large pits, total pits, fine scratches, coarse scratches, and total scratches)

		Sum of sqrs	df	Mean sqrs	F	p
Small pits	Between groups:	62.371	3	20.7903	6.583	**0.002102**
	Within groups:	75.7993	24	3.1583		
	Total:	138.17	27			
Large pits	Between groups:	151.206	3	50.4018	10.72	**0.000116**
	Within groups:	112.844	24	4.70182		
	Total:	264.049	27			
Total pits	Between groups:	262.158	3	87.3859	9.727	**0.00022**
	Within groups:	215.614	24	8.98391		
	Total:	477.772	27			
Fine scratches	Between groups:	10.0194	3	3.33979	0.8671	0.4717
	Within groups:	92.4437	24	3.85182		
	Total:	102.463	27			
Coarse scratches	Between groups:	53.8274	3	17.9425	5.46	**0.005258**
	Within groups:	78.875	24	3.28646		
	Total:	132.702	27			
Total scratches	Between groups:	105.211	3	35.0702	4.214	**0.01577**
	Within groups:	199.719	24	8.32161		
	Total:	304.929	27			

Significant *p*-values are in bold

The bivariate plot compares mean values of total number of fine and coarse scratches (TS) versus total number of small and large pits (TP) (Fig. 1) for all five analyzed taxa. Both *Orophodon* and the two teeth of *Orophodon* vel *Octodontotherium* have similar numbers of pits of any kind compared with *Octodontotherium* but cluster well apart from the latter towards higher numbers of scratches of any kind. Regarding total numbers of pits, all three fossil sloths fall in the gap between *Choloepus* and *Bradypus*, with the former showing lower values (mainly due to significantly higher numbers of small pits) and the latter showing larger values (mainly due to significantly higher numbers of large pits). A principal component analysis (PCA) was run on a correlation matrix including raw values of those use wear parameters which showed to be significantly different between taxa: number of small pits; number of large pits; number of coarse scratches; and presence/absence of puncture pits. The first principal component explained about 59% of the total variance. The second component explained about one-quarter of the total variance. Therefore, we regarded the subsequent components as insignificant in terms of their contribution to the total variance. The loading of each variable of the PCA on the components is shown in Table 5, the resulting scatter plot with convex hulls embracing the areas occupied by the each taxon is shown in Fig. 2.

**Table 5:** Loadings of each variable of the PCA on the components showing significant differences (stereomicroscopic microwear)

	PC 1	PC 2	PC 3
SP	0.56524	0.45958	0.32948
LP	**0.72561**	−0.38378	−0.03028
CS	0.47668	0.59748	−0.31463
PP	−0.02628	**0.89656**	0.15955

Loadings in bold are above cut-off (+/− 0.7)

PCA1 shows high positive loadings for the number of large pits. With the highest values, *Bradypus* plots on the right end of the graph, while *Choloepus*, having the lowest values, plots on the left end. There is a minute overlap of areas of *Octodontotherium* and *Choloepus* as well as of *Orophodon* and *Bradypus*. The areas occupied by *Octodontotherium* and *Orophodon* overlap largely showing similar values for the number of large pits.

PCA2 is highly influenced by the positive loading of the presence/absence of puncture pits. All fossil sloths individuals showed puncture pits whereas puncture pits were present in one out of eight individuals in *B. variegatus* (12.5%) and one out of six individuals of *C. didactylus* (33%).

### SEM Microwear

**Fig. 3:**
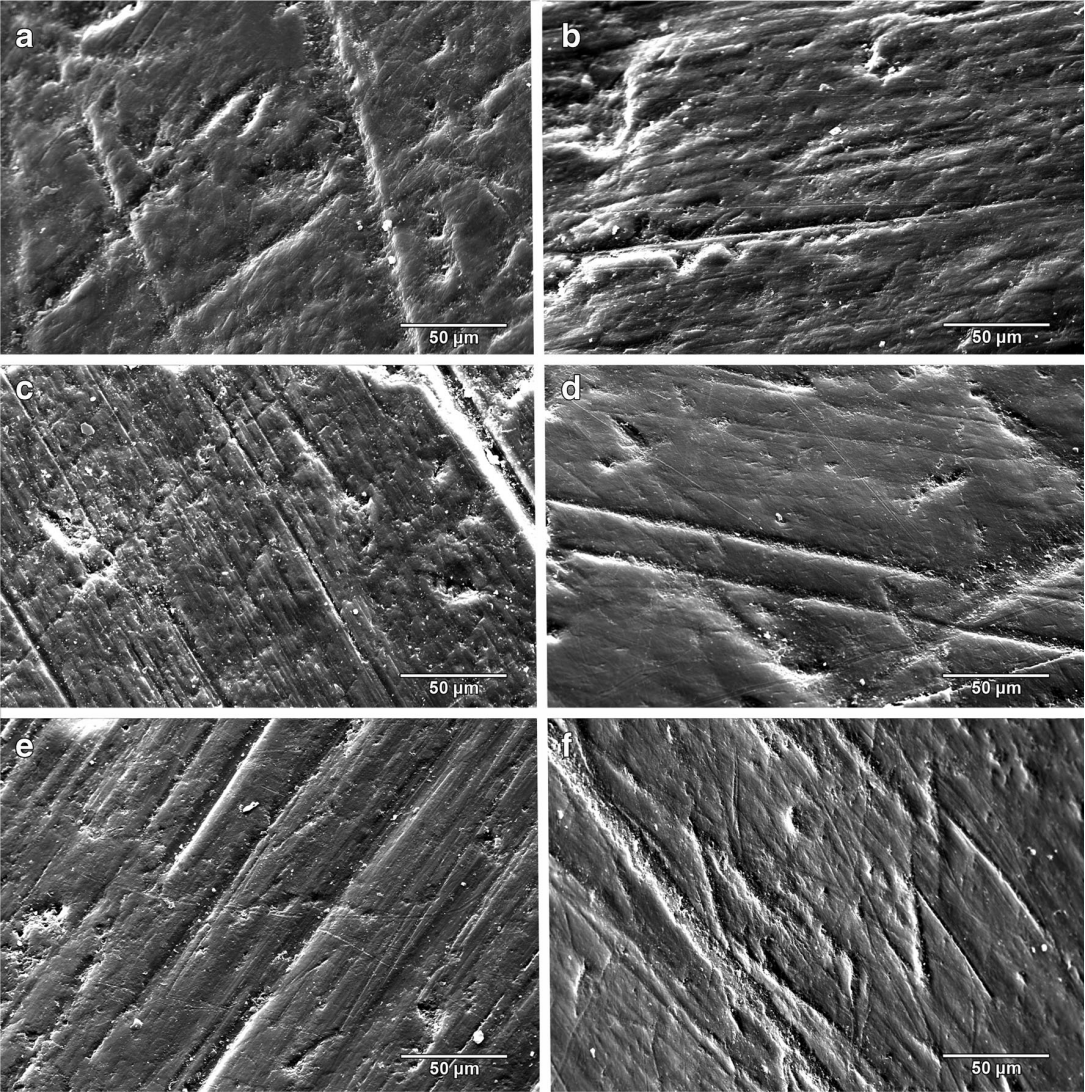
Representative SEM images of orthodentine microwear from the three analyzed taxa of Oligocene sloths. **a-c,** *Octodontotherium grande*. **a,** Right upper molariform 4 (MNHN DES 238); **b,** Left upper molariform 4 (MNHN DES 237); **c,** Left upper molariform 5 (MNHN DES 245). **d,** *Orophodon hapaloides* vel *Octodontotherium grande*, lower molariform ?2 (MNHN DES 233). **e-f,** *Orophodon hapaloides*. **e,** Right lower molariform 4 (MNHN DES 267); **f,** Left upper molariform 3 or 4 (MNHN DES 269). MNHN = Musée National d’Histoire Naturelle, Paris (France)

**Fig. 4:**
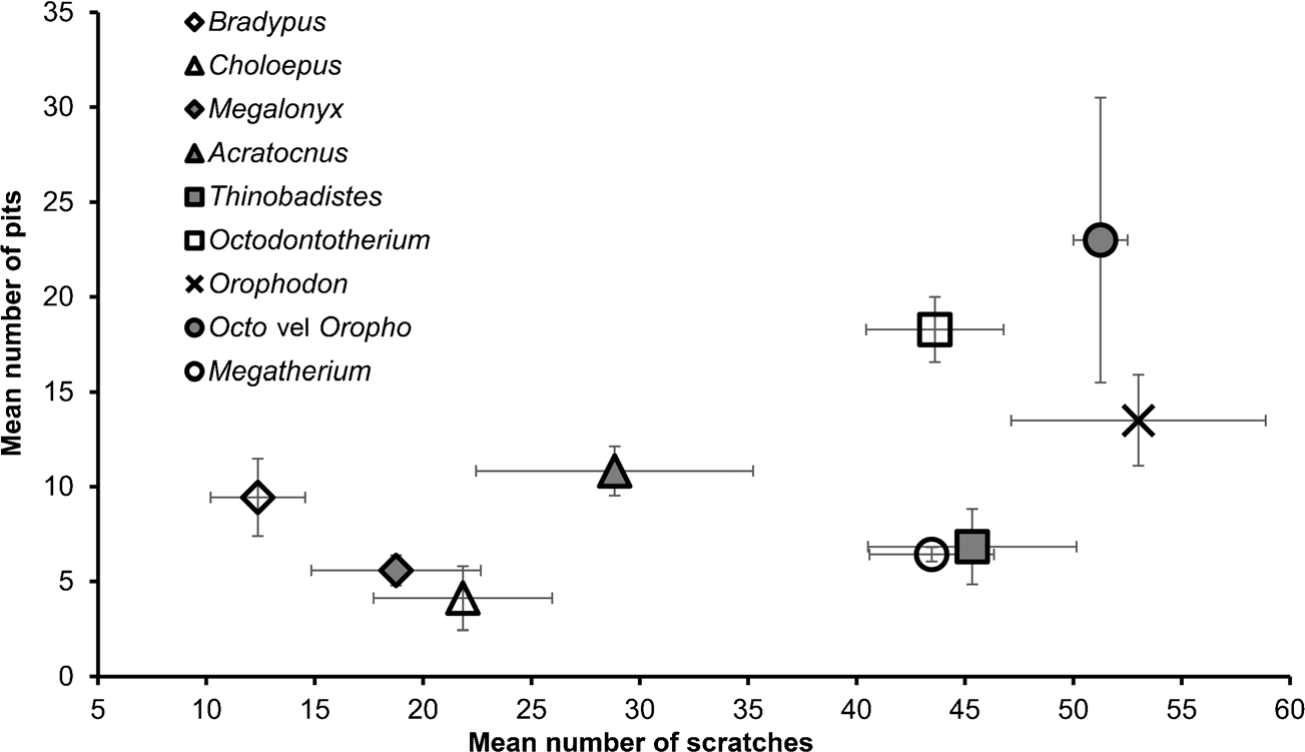
Bivariate plot comparing mean number of scratches versus mean number of pits for all taxa analyzed by SEM microwear. Data for *Acratocnus odontrigonus*, *Bradypus variegatus*, *Choloepus* sp. *Megalonyx wheatleyi*, *Megatherium americanum*, and *Thinobadistes segnis* are taken from Resar et al. (2013) and Green and Kalthoff (2015)

**Fig. 5:**
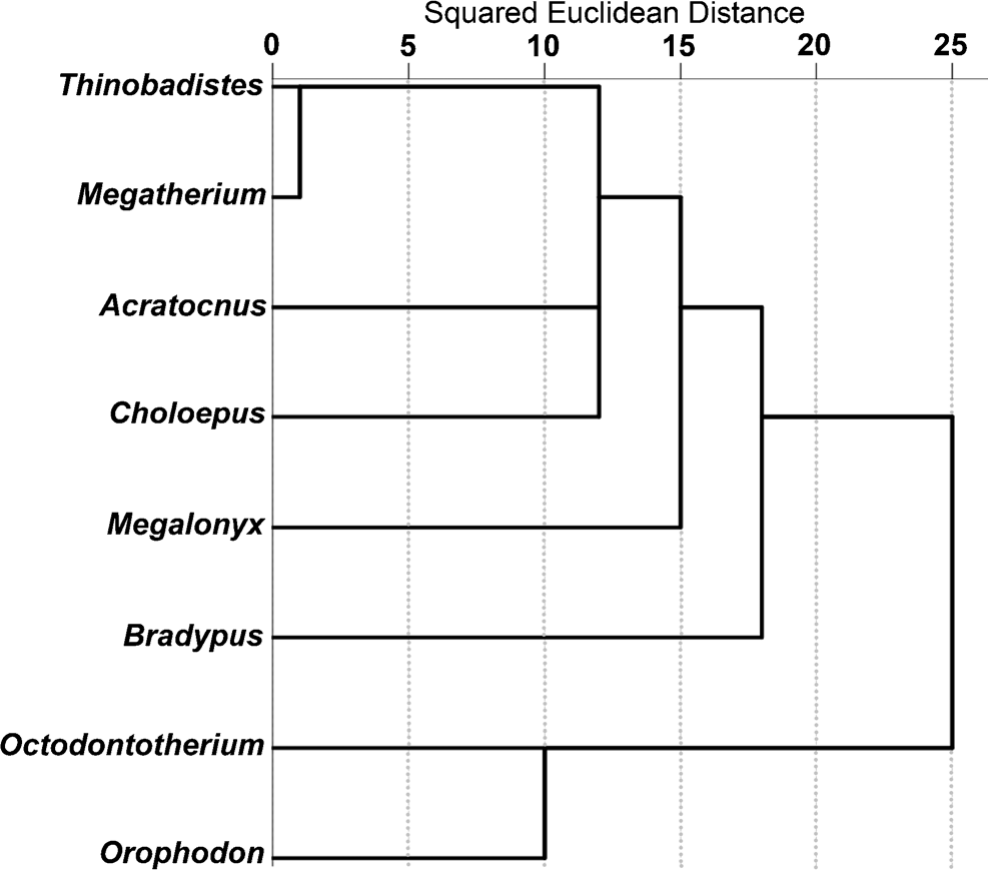
Hierarchical cluster analysis showing squared Euclidean distances among all taxa analyzed by SEM microwear. Data for *Acratocnus odontrigonus*, *Bradypus variegatus*, *Choloepus* sp. *Megalonyx wheatleyi*, *Megatherium americanum*, and *Thinobadistes segnis* are taken from Resar et al. (2013) and Green and Kalthoff (2015)

**Fig. 6:**
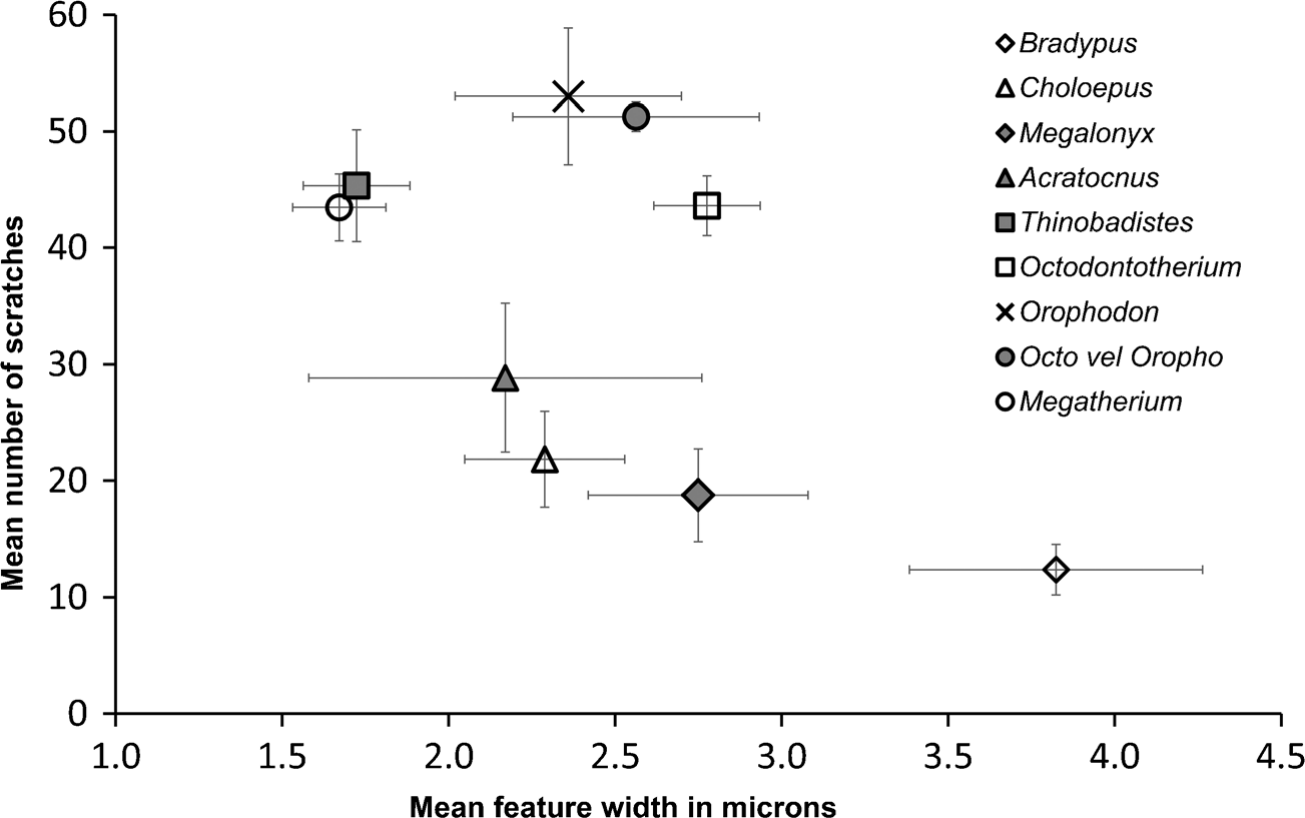
Bivariate plot comparing mean feature width versus mean number of scratches for all taxa analyzed by SEM microwear. Data for *Acratocnus odontrigonus*, *Bradypus variegatus*, *Choloepus* sp. *Megalonyx wheatleyi*, *Megatherium americanum*, and *Thinobadistes segnis* are taken from Resar et al. (2013) and Green and Kalthoff (2015)

Microwear results are summarized in Table 6; raw counts are included in Supplementary Table 3. There were no significant differences in any variable for the ten replicate images (S: Z = −0.77, *p* = 0.44; P: Z = −1.80, *p* = 0.07; FW: Z = −0.56, *p* = 0.58; R: Z = −0.05, *p* = 0.96), so the observer was able to consistently replicate feature counts on the same images. At high magnification, wear surfaces on Oligocene sloth teeth are dominated by a unique combination of high scratch and pit counts compared to previously sampled sloths (Figs. 3, 4; Table 6). This relative difference in microwear signature is further supported by cluster analysis (Fig. 5). However, the pair-wise differences between species were not always significant. The number of scratches was normally distributed (*t* = 0.99, df = 50, *p* = 0.94) with equal variance (Levene statistic = 0.63, df = 7.42, *p* = 0.73). The only significant difference in mean S was between *Orophodon* and *Choloepus*, with the former having a significantly higher scratch count (Table 7). All other variables had non-normal distributions (FW: *t* = 0.90, df = 50, *p* < 0.01; P: *t* = 0.89, df = 50, *p* < 0.01; R: *t* = 0.93, df = 50, *p* < 0.01). No significant differences among species were present for R (Table 8). Both *Thinobadistes* and *Megatherium* have a significantly lower FW than *Bradypus*, while *Octodontotherium* has a significantly higher number of pits compared to *Megatherium*, *Bradypus*, and *Thinobadistes* (Figs. 4, 6; Table 8).

**Table 6:** Mean SEM microwear variables for all individuals, with species mean and standard deviation

Specimen	Tooth	FW (μm)	R	Pits	Scratches
*Octodontotherium grande*					
MNHN-DES 236	Rmf4	2.84	0.30	27.50	46.00
MNHN-DES 237	LMF4	2.45	0.68	23.00	41.00
MNHN-DES 238	RMF4	3.49	0.52	16.50	39.50
MNHN-DES 239	Lmf3	2.92	0.48	20.00	35.00
MNHN-DES 245	LMF5	2.22	0.46	18.50	54.00
MNHN-DES 246	Lmf4	2.86	0.24	16.00	53.50
MNHN-DES 250	LMF4	2.84	0.27	18.50	39.50
MNHN-DES 251	M/mf?	3.61	0.40	15.50	28.00
MNHN-DES 252	RMF4	1.78	0.61	9.00	56.00
Mean		**2.78**	**0.44**	**18.28**	**43.61**
St Dev		**0.58**	**0.15**	**5.15**	**9.51**
*Orophodon hapaloides*					
MNHN-DES 260	mf1/mf2	1.16	0.15	5.00	69.50
MNHN-DES 267	Rmf4	2.30	0.31	13.50	54.00
MNHN-DES 268	RMF2	3.28	0.21	19.50	47.50
MNHN-DES 269	LMF3/MF4	2.40	0.76	16.00	59.50
MNHN-DES 277	Lmf4	2.67	0.50	13.50	34.50
Mean		**2.36**	**0.38**	**13.50**	**53.00**
St Dev		**0.77**	**0.25**	**5.35**	**13.11**
*Octodontotherium* vel *Orophodon*					
MNHN-DES 233	mf2?	2.20	0.47	15.50	52.50
MNHN-DES 234	mf2?	2.93	0.53	30.50	50.00
Mean		**2.56**	**0.50**	**23.00**	**51.25**
St Dev		**0.52**	**0.04**	**10.61**	**1.77**

The values bolder here reflect mean and standard variation

Molariforms from the maxilla are denoted by MF, those from the mandible by mf; L = left, R = right

Abbreviations: *FW* mean width of all features, *R* relative orientation of features, with 0 representing random orientation and 1 equaling perfectly aligned features on tooth surface, *Pits* mean number of pits, *Scratches* mean number of scratches

**Table 7:** Results of One-way ANOVA and Levene’s test for homogeneity of variance for scratches, with *p*-values from Tukey HSD post-hoc tests (SEM microwear)

	Test stat	df	p					
ANOVA	2.43	49	**0.04**					
Levene’s	0.63	7. 42	0.73					
Tukey HSD	*Acratocnus*	*Megalonyx*	*Thinobadistes*	*Bradypus*	*Choloepus*	*Megatherium*	*Octodonto-therium*	*Orophodon*
*Acratocnus*		0.99	1.00	0.86	0.37	0.99	0.99	0.99
*Megalonyx*	0.99		0.98	0.97	0.49	1.00	1.00	0.75
*Thinobadistes*	1.00	0.98		0.52	0.09	0.98	0.97	0.99
*Bradypus*	0.86	0.97	0.52		0.94	0.97	0.95	0.17
*Choloepus*	0.37	0.49	0.10	0.94		0.46	0.37	**0.02**
*Megatherium*	0.99	1.00	0.97	0.97	0.46		1.00	0.71
*Octodontotherium*	0.99	1.00	0.97	0.95	0.37	1.00		0.67
*Orophodon*	0.99	0.75	0.99	0.17	**0.02**	0.71	0.67	

Data for *Acratocnus odontrigonus*, *Bradypus variegatus, Choloepus* sp. *Megalonyx wheatleyi*, *Megatherium americanum*, and *Thinobadistes segnis* are taken from Resar et al. (2013) and Green and Kalthoff (2015)

Significant *p*-values are in bold

**Table 8:** Results of Kruskal-Wallis test for FW, R, and Pits (not normally distributed) with Dunn-Bonferroni post-hoc tests for variables with significant differences (SEM microwear)

Kruskal-Wallis	t	df	p					
FW	28.88	7	**<0.01**					
R	9.95	7	0.19					
Pits	28.88	7	**<0.01**					
Dunn-Bonferroni post-hoc tests								
FW	*Acratocnus*	*Megalonyx*	*Thinobadistes*	*Bradypus*	*Choloepus*	*Megatherium*	*Octodontotherium*	*Orophodon*
*Acratocnus*		1.00	1.00	0.92	1.00	1.00	1.00	1.00
*Megalonyx*	1.00		0.56	1.00	1.00	0.36	1.00	1.00
*Thinobadistes*	1.00	0.56		**0.01**	1.00	1.00	0.27	1.00
*Bradypus*	0.92	1.00	**0.01**		0.56	**<0.01**	1.00	1.00
*Choloepus*	1.00	1.00	1.00	0.56		1.00	1.00	1.00
*Megatherium*	1.00	0.36	1.00	**<0.01**	1.00		0.15	1.00
*Octodontotherium*	1.00	1.00	0.27	1.00	1.00	0.15		1.00
*Orophodon*	1.00	1.00	1.00	1.00	1.00	1.00	1.00	
Pits	*Acratocnus*	*Megalonyx*	*Thinobadistes*	*Bradypus*	*Choloepus*	*Megatherium*	*Octodontotherium*	*Orophodon*
*Acratocnus*		1.00	1.00	1.00	1.00	1.00	1.00	1.00
*Megalonyx*	1.00		1.00	1.00	1.00	1.00	0.17	1.00
*Thinobadistes*	1.00	1.00		1.00	1.00	1.00	**<0.01**	0.65
*Bradypus*	1.00	1.00	1.00		1.00	1.00	**<0.01**	0.12
*Choloepus*	1.00	1.00	1.00	1.00		1.00	0.26	1.00
*Megatherium*	1.00	1.00	1.00	1.00	1.00		**0.02**	1.00
*Octodontotherium*	1.00	0.17	**<0.01**	**<0.01**	0.26	**0.02**		1.00
*Orophodon*	1.00	1.00	0.65	0.12	1.00	1.00	1.00	

Data for *Acratocnus odontrigonus*, *Bradypus variegatus, Choloepus* sp. *Megalonyx wheatleyi*, *Megatherium americanum*, and *Thinobadistes segnis* are taken from Resar et al. (2013) and Green and Kalthoff (2015)

Significant *p*-values are in bold

## Discussion

Independently, both stereomicroscopic microwear (low-magnification) and SEM microwear (high-magnification) tell a similar “story,” whereby Oligocene sloths are clearly different than extant sloths. However, the quantitative variables analyzed and relative abundance of features observed is different between the two methods. For example, stereomicroscopy revealed a lower absolute number of total scratches (<20) at 70× for *Octodontotherium* and *Orophodon*, whereas SEM counts for the same specimens had scratch densities that were >40. This difference in absolute feature densities is not surprising due to the differences in 1) imaging and counting technique, and 2) magnification level. Light microscopy relies on refraction of an external light source to illuminate features, and the user has to identify features directly through the lens. On the other hand, SEM relies on electron saturation and detection to form a high resolution digital image and the user counts features on a computer screen. Scratches and pits will thus be recognized and identified in a different manner between the techniques (Solounias and Semprebon 2002). Additionally, and perhaps more importantly, the same surface viewed at 500× will have a significantly higher density of features visible compared to the same surface at 70× (Gordon 1988; Green and Croft in press). At lower levels, the finest features will not even be perceptible to the human eye, whereas at 500×, scratches that measure <1 μm in width are easily identifiable (Fig. 3). Indeed, the mean width of scratches seen under SEM for Oligocene sloths was <1.60 μm (pers. obs., JLG); these fine features are not visible at 70×. Additionally, some coarse features seen under stereomicroscopy (e.g., large puncture pits, hypercoarse scratches) can have widths >100 μm (Semprebon et al. 2004) and the boundaries of such large features would extend beyond the boundary of field of view at 500× under SEM; thus, they cannot be scored via SEM. For these reasons, our goal was not to reproduce absolute feature counts between the two methods, as such is neither possible nor necessary. In user-based microwear methods, relative differences in quantitative variables among taxa is more informative than relying on absolute benchmarks to define dietary differences (Mihlbachler et al. 2012). Thus, below we interpret the results from the two techniques separately (in the context of relative differences to other sloths within each technique) and then combine our separate interpretations to hypothesize the feeding ecology of *Octodontotherium* and *Orophodon*.

### Stereomicroscopic Microwear

**Fig. 7:**
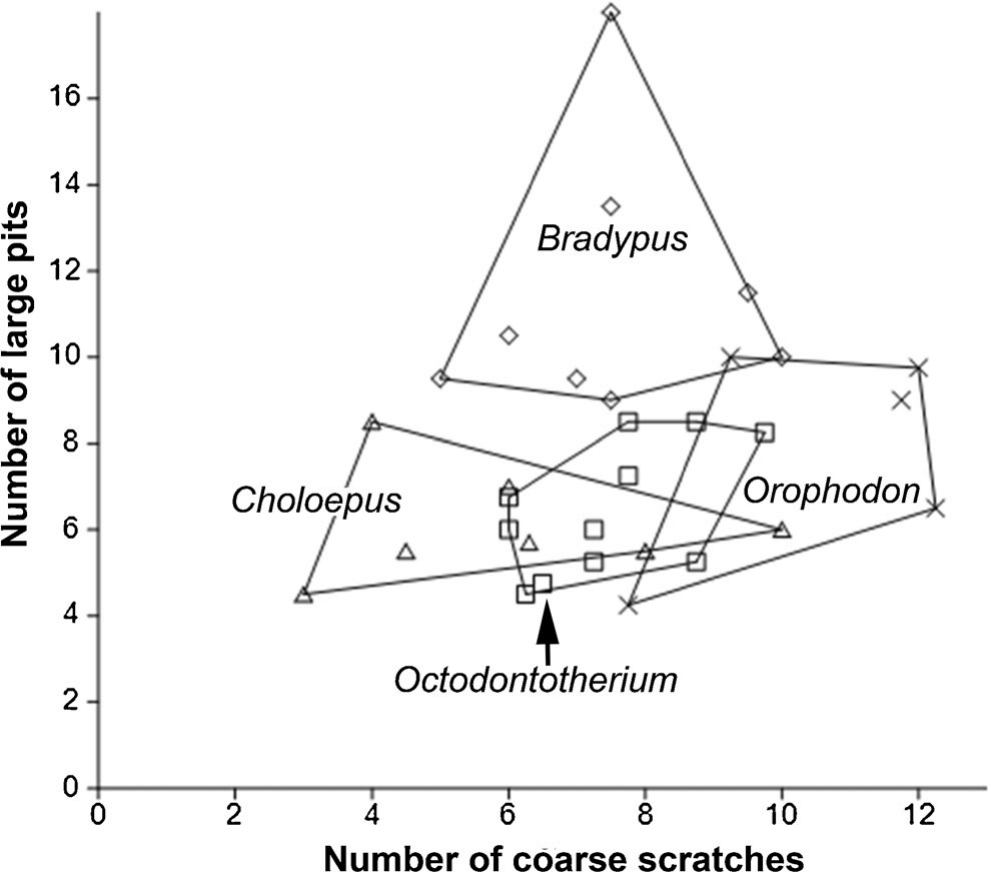
Bivariate plot comparing raw values of number of coarse scratches (CS) versus number of large pits (LP). Convex hulls embrace the areas taken by four taxa analyzed by stereomicroscopic microwear

Comparing results for *Octodontotherium* and *Orophodon* feeding adaptations appear to have been rather similar in these two taxa: the areas embraced by convex hulls are largely overlapping in bivariate plots of raw values of TS versus TP (not figured). However, when comparing only the coarse features (CS versus LP, Fig. 7), *Orophodon* clearly clusters towards higher values of CS (whose counts are significantly different from *Octodontotherium*) suggesting ingestion of somewhat tougher food items. Averaged values for the two specimens of *Octodontotherium* vel *Orophodon* cluster close to *Orophodon* in the bivariate plot TS versus TP (Fig. 1).

The extant folivore *Bradypus variegatus* separates well from the frugivore–folivore *Choloepus didactylus* (dietary categories after Green 2009a and references therein) in respect to all three pit parameters (SP, LP, TP) with no overlap in raw values of LP and of TP and very minor overlap in SP. The fossil sloths fall in the gap between the low pit counts of *Choloepus* and the high pits counts in *Bradypus* but show on average higher scratch values (Figs. 1 and 7). Fossil sloths likewise fall between the extant species with moderate to low numbers of LP for PCA 1, but fall towards high values for PP on the PCA2 axis (Table 5; Fig. 2). We conclude that feeding ecology in the Oligocene sloths was different from that of the extant species.

Moderate to high numbers of pits of all kinds suggest consumption of large quantities of leaves and fruit pulp, whereas twigs or seeds/fruits may account for frequent CS, including hypercoarse scratches. However, a high number of CS were characteristic for the enamel and orthodentine areas in teeth of the grazing rhinoceros *Ceratotherium simum* and significantly distinguishes it from the other four rhinoceros species (pers. obs., DCK). *Ceratotherium simum* is a bulk feeder foraging at ground level almost exclusively on abrasive dry grasses in open habitats (e.g., Groves 1972; Estes 1991; Mendoza et al. 2002). Even though tooth morphology and architecture are very different in the White rhinoceros and the Oligocene sloths, this comparison allows for the consideration that consumption of abrasive food like grass have been of certain importance, especially in *Orophodon*. However, if the habitat assumption (“emerging savannas and grasslands”) of Shockey and Anaya (2011) is correct, we expect an environmental influence of simultaneously ingested dust and grit on microwear features such as coarse scratches and gouging (see below).

The presence of PP in all analyzed teeth of *Octodontotherium* and *Orophodon* can be interpreted as seed predation in the form of grass spikelets and/or from seed-rich fruit. Larger-sized PP and smaller PP with associated coarse scratches were seen in several teeth and can be interpreted as seed indentation (from grass and/or fruit) with subsequent scratching during mastication. Frequent gouging on tooth surfaces of the fossil species suggests that extrinsic factors, such as possible intake of abrasive grit by feeding on ground-level vegetation, may be influencing tooth wear. However, frequent gouging is also present in the analyzed extant sloths which are high-canopy forest dwellers.

The question remains whether *Octodontotheriun* and *Orophodon* were (1) anatomically capable to execute such movements to reach the levels of the here predicted food sources (ground level, bushes, trees), and (2) whether their teeth and their craniodental morphology allowed consumption of abrasive and or/or hard food items. Firstly, a small number of postcranial skeletal elements such as astragali, a humerus fragment, some elements of the carpus, metapodials, and phalanges were attributed to *Octodontotherium*, *Orophodon*, and their close relative *Paroctodontotherium calleorum* on the basis of finding association and size (Hoffstetter 1956; Shockey and Anaya 2011). These remains do not offer sufficient evidence to infer locomotor abilities in these taxa. However, when taking mylodontid ground sloths as a group into account, postcranial remains from well-documented taxa such as the Lujanian (late Pleistocene/early Holocene) *Glossotherium* and *Scelidotherium*, show shorter forelimbs than hind limbs suggesting a quadruped, somewhat forward inclined natural posture (Bargo et al. 2000). In addition, the authors model the center of mass “almost perpendicular above the anterior parts of the hind feet” (Bargo et al. 2000:604), which implies that these animals were most probably capable to bipedal stances. With these prerequisites, mylodontids are expected to have the anatomical requirements for exploiting food sources from ground to tree level. Secondly, as all ground sloths, *Octodontotheriun* and *Orophodon* have hypselodont teeth that are capable of coping with tissue loss caused by periodic feeding on abrasive and or/or hard food items (Shockey and Anaya 2011). The only preserved skull belongs to *Octodontotherium* (Shockey and Anaya 2011:fig. 2b) and features a wide muzzle that is largely comparable to that of the Lujanian *Glossotherium robustum* (Bargo et al. 2006). The authors conclude that in ground sloths, wide muzzles are found in parallel with a nonprehensile lip and a movable tongue and that these features characterize them as bulk feeders at ground level making them “best-adapted …to a grazing niche” Bargo et al. 2006:261).

In summary, *Octodontotherium* and *Orophodon* show a high variation of microwear features indicating a diversified diet of food with low intrinsic toughness (i.e., soft foliage, fruit pulp) to high intrinsic toughness (i.e., seeds/fruits) as well as food items with moderate to high abrasiveness (i.e., grass, twigs, tough foliage). Given that the deduction of ingested food items (foliage, grass, fruit) we made from the microwear features is correct, this varied menu would be an argument for bulk feeding in the analyzed Oligocene sloth species, an alimentary style which is supported by craniodental features. Feeding is expected to have occurred both at higher levels (foliage, twigs, and fruit) as well as at ground level (grass, fallen fruit). Frequent gouging and high numbers of coarse scratches account for a certain influence of extrinsic factors on microwear results, such as contamination of food items with grit and dust.

### Microwear Analysis with SEM

When microwear patterns from living and extinct sloths are compared to our new data, *Octodontotherium* and *Orophodon* cluster in a distinct grouping from all other taxa sampled to date. Specifically, scratch and pit counts are the SEM variables that tend to quantitatively distinguish these sloths.

As with enamel, orthodentine microwear corresponds to both endogenous (e.g., hardness, toughness of food items) and exogenous factors (e.g., abrasive grit) during mastication. Microwear patterns in living sloths are interpreted to represent mainly endogenous factors, as the influence of abrasive grit on food items in the tropical canopy is less than that closer to ground level (Green and Resar 2012). Among tree sloths, a strict folivorous diet generally correlates with greater feature width and lower scratch counts compared to a more general frugivorous-folivorous one (Green and Resar 2012). Although *Octodontotherium* and *Orophodon* have a mean feature width that is comparable to *Choloepus* but not *Bradypus* (Fig. 6), scratch counts are always higher in Oligocene taxa compared to both tree sloths (significantly so in *Orophodon* vs. *Choloepus*; Table 7; Fig. 6). These relative differences suggest that Oligocene sloths had a different diet than living sloths. Among other extinct sloths, *Megatherium* and *Thinobadistes* also show significant differences from *Bradypus* in terms of feature width (Table 8). Oligocene sloths always have a higher mean pit count (significantly so for *Octodontotherium* versus *Bradypus*, *Thinobadistes*, and *Megatherium*) than other sloths. Our findings here represent the first time that number of pits has differed significantly among sloths in any microwear analysis to date (Green 2009a; Green and Resar 2012; Resar et al. 2013; Green and Kalthoff 2015). When viewed collectively, it is most likely the combination of a relatively high mean scratch and pit count that distinguishes *Octodontotherium* and *Orophodon*. This suggests that these Oligocene taxa had a different feeding ecology, compared to other sloths sampled to date (Fig. 5). For enamel microwear, a higher scratch density has been correlated to increased chewing cycles to process tough food items (e.g., tough leaves, grass), whereas higher pit density is related to feeding on more hard-objects (e.g., seeds) (Teaford and Walker 1984; Teaford 1988; Solounias and Semprebon 2002). If we assume the same food texture to microwear relationship exists in orthodentine, then it appears that *Octodontotherium* and *Orophodon* are feeding on a combination of very tough and hard foods, leading to a higher density of both scratches and pits on their teeth. However, enamel is a much harder dental tissue and thus less likely scar as easily as orthodentine, which means that food items would not have to be as hard and/or tough to significantly scar sloth teeth (Lucas 2004). An environmental influence of abrasive grit on high scratch density has been suggested for *Megatherium* and *Thinobadistes* (Green and Kalthoff 2015), yet these taxa did not have high pit counts. Thus, if there is a significant exogenous influence on microwear for *Octodontotherium* and *Orophodon*, it is different from that influencing other ground sloths. Feeding on a high amount of abrasive grit (a “hard object” by definition) would likely also cause heavy pitting. Given these consideration, we hypothesize that SEM microwear reflects *Octodontotherium* and *Orophodon* feeding on moderately-tough vegetation at ground level. However, this hypothesis remains to be tested further as our understanding of how different foods create discrete microwear patterns on orthodentine improves.

## Conclusions

Whether analyzed via low-magnification stereomicroscopy (coarse scale) or high-magnification SEM (fine scale), microwear patterns in fossil *Octodontotherium grande*, *Orophodon hapaloides*, and *Orophodon* vel *Octodontotherium* are unlike those found in extant *Bradypus* and *Choloepus*. Thus, our initial hypothesis 1 is supported by both techniques. For the SEM analysis, the Oligocene taxa sampled here in general have a unique combination of high scratch and pit counts. Thus, Oligocene basal mylodontoids had a different feeding ecology than more derived mylodontid and megatheroid sloths analyzed to date (meaning rejection of hypothesis 2).

Collectively, the relative differences among variables suggest that these basal Oligocene mylodontoids fed on plant material with at least moderate intrinsic toughness (foliage, twigs). A rather rough and grained occlusal surface discernable at the microscopic level (which surprisingly is in sharp contrast to its smooth macro-optical and tactile appearance, also mentioned by Shockey and Anaya (2011) for *Paroctodontotherium*) indicates that both taxa also included more brittle, hard objects (e.g., seeds) in their diet, which is supported by the high frequency of puncture pits in stereomicroscopy and an elevated overall number of pits in SEM. Higher numbers of coarse scratches, comparable with results from the grazing rhinoceros *Ceratotherium simum* (pers. obs., DCK), might account for similar feeding preferences, especially for *Orophodon*. The elevated presence of pits on tooth surfaces also suggests that extrinsic factors, such as possible intake of abrasive grit by feeding on ground level vegetation, may be influencing tooth wear. The overall picture suggests a highly varied, herbivorous diet for the Oligocene sloths exploiting diverse food resources of their habitat.

These interpretations generally support Shockey and Anaya (2011) in reconstructing (1) both taxa as wide-muzzled bulk feeders of plants with moderate intrinsic toughness at ground level and/or at higher levels, and (2) Deseadan environments as developing open habitats with spreading savanna-like vegetation. Bulk-feeding at ground level might include grazing. As a result, grazing in these Oligocene sloths should not be eliminated as a possible part of their diet, but neither can it be directly supported, as no living xenarthran is a grazer and thus there is currently no basis for identifying a “grazing” microwear signature on orthodentine, at least without other supporting lines of evidence. Recently, Saarinen and Karme (2017) published an analysis that successfully used the angle of dental mesowear as proxy for diet in xenarthrans. A similar mesowear angle between the Oligocene sloths sampled here and hypothesized grazers like *Scelidotherium* and *Lestodon* (Saarinen and Karme 2017) would support a grazing diet in the former. As our understanding of how food texture creates distinct microwear features on orthodentine improves, so will our interpretation of the specific diet of these ancestral sloths.

These interpretations show as well that both applied microwear techniques, coarse scale low-magnification stereomicroscopy and fine scale high-magnification SEM, came to congruent conclusions regarding feeding ecology in the Oligocene mylodontoid sloths *Octodontotherium grande* and *Orophodon hapaloides*.

## Electronic supplementary material

ESM 1 (DOCX 23 kb)

ESM 2 (DOCX 19 kb)

ESM 3 (DOCX 19 kb)
