# Supplementary material for: Feeding Ecology in Oligocene Mylodontoid Sloths (Mammalia, Xenarthra) as Revealed by Orthodentine Microwear Analysis
Source: J Mamm Evol. 2017 Jul 28;25(4):551–64. doi: 10.1007/s10914-017-9405-x (PMC6209052; doi:10.1007/s10914-017-9405-x)
Supplement: Supplementary file 1 — (DOCX 23 kb) [file 10914_2017_9405_MOESM1_ESM.docx]

Feeding ecology in Oligocene mylodontoid sloths (Mammalia, Xenarthra) as revealed by orthodentine microwear analysis. Journal of Mammalian Evolution. Kalthoff DC*, Green JL

Department of Zoology, Swedish Museum of Natural History, Box 50007, SE–104 05 Stockholm, Sweden (DCK)

Department of Geology, Kent State University at Tuscarawas, 330 University Drive NE, New Philadelphia, OH 44663, USA (JLG)

* Corresponding author: Kalthoff DC, E-Mail: daniela.kalthoff@nrm.se

**Supplementary Table 1**

Raw stereoscopic microwear variables for all fossil individuals. Four separate countings were executed per specimen, designated by a 1, 2, 3 or 4 following the specimen number. Abbreviations: SP = number of small pits; LP = number of large pits; FS = number of fine scratches; CS = number of coarse scratches; G = gouges; PP = puncture pits; 0 = feature absent; 1 = feature present. MNHN = Musée National d’Histore Naturelle, Paris, France.

| **Specimen** | **SP-1** | **SP-2** | **SP-3** | **SP-4** | **LP-1** | **LP-2** | **LP-3** | **LP-4** | **FS-1** | **FS-2** | **FS-3** | **FS-4** | **CS-1** | **CS-2** | **CS-3** | **CS-4** | **G-1** | **G-2** | **G-3** | **G-4** | **PP-1** | **PP-2** | **PP-3** | **PP-4** |
| --- | --- | --- | --- | --- | --- | --- | --- | --- | --- | --- | --- | --- | --- | --- | --- | --- | --- | --- | --- | --- | --- | --- | --- | --- |
| *Octodontotherium grande*  MNHN-DES 236 | 9 | 10 | 10 | 8 | 6 | 6 | 4 | 5 | 12 | 11 | 10 | 8 | 10 | 8 | 6 | 11 | 1 | 1 | 1 | 1 | 0 | 1 | 1 | 0 |
| MNHN-DES 237 | 13 | 15 | 15 | 11 | 4 | 4 | 8 | 2 | 5 | 6 | 8 | 9 | 9 | 5 | 3 | 8 | 1 | 1 | 1 | 1 | 1 | 1 | 1 | 1 |
| MNHN-DES 238 | 11 | 11 | 15 | 14 | 7 | 6 | 12 | 8 | 15 | 7 | 12 | 12 | 14 | 13 | 8 | 4 | 0 | 0 | 0 | 0 | 1 | 1 | 0 | 0 |
| MNHN-DES 239 | 16 | 17 | 15 | 12 | 7 | 6 | 5 | 6 | 7 | 8 | 10 | 8 | 3 | 7 | 9 | 10 | 0 | 1 | 1 | 1 | 1 | 0 | 1 | 1 |
| MNHN-DES 245 | 11 | 13 | 12 | 12 | 9 | 10 | 11 | 4 | 9 | 7 | 9 | 6 | 5 | 13 | 11 | 6 | 1 | 1 | 1 | 0 | 1 | 0 | 0 | 0 |
| MNHN-DES 246 | 11 | 10 | 7 | 14 | 5 | 5 | 4 | 10 | 4 | 5 | 8 | 12 | 7 | 9 | 3 | 5 | 0 | 0 | 1 | 1 | 0 | 0 | 1 | 0 |
| MNHN-DES 250 | 14 | 14 | 10 | 16 | 10 | 7 | 6 | 11 | 14 | 6 | 11 | 8 | 10 | 7 | 7 | 7 | 1 | 1 | 1 | 1 | 1 | 0 | 1 | 1 |
| MNHN-DES 251 | 9 | 8 | 15 | 5 | 5 | 5 | 6 | 5 | 8 | 8 | 5 | 4 | 9 | 5 | 6 | 9 | 1 | 1 | 0 | 1 | 1 | 0 | 1 | 1 |
| MNHN-DES 252 | 14 | 12 | 11 | 10 | 4 | 7 | 5 | 3 | 12 | 7 | 8 | 4 | 5 | 8 | 8 | 5 | 1 | 0 | 1 | 1 | 0 | 0 | 1 | 0 |
| *Orophodon hapaloides*  NHN-DES 260 | 15 | 23 | 14 | 10 | 9 | 14 | 5 | 8 | 11 | 13 | 11 | 14 | 14 | 11 | 8 | 14 | 0 | 0 | 1 | 1 | 1 | 0 | 1 | 1 |
| MNHN-DES 267 | 10 | 11 | 15 | 13 | 6 | 10 | 6 | 4 | 9 | 15 | 10 | 10 | 12 | 17 | 12 | 8 | 0 | 1 | 1 | 1 | 0 | 0 | 0 | 1 |
| MNHN-DES 268 | 7 | 15 | 8 | 12 | 6 | 6 | 1 | 4 | 8 | 5 | 8 | 7 | 6 | 10 | 7 | 8 | 0 | 0 | 0 | 0 | 1 | 0 | 1 | 1 |
| MNHN-DES 269 | 11 | 8 | 8 | 11 | 12 | 9 | 9 | 10 | 11 | 8 | 6 | 8 | 5 | 12 | 9 | 12 | 0 | 0 | 0 | 0 | 1 | 1 | 1 | 1 |
| MNHN-DES 277 | 8 | 12 | 14 | 15 | 9 | 6 | 9 | 15 | 12 | 7 | 6 | 5 | 13 | 12 | 14 | 9 | 0 | 1 | 1 | 1 | 1 | 1 | 1 | 0 |
| *Octodontotherium vel Orophodon* | | | | | | | | | | | | | | | | | | | | | | | | |
| MNHN-DES 233 | 11 | 11 | 11 | 12 | 8 | 4 | 2 | 4 | 8 | 10 | 9 | 4 | 4 | 4 | 7 | 5 | 1 | 1 | 0 | 0 | 1 | 1 | 1 | 1 |
| MNHN-DES 234 | 15 | 12 | 15 | 14 | 9 | 5 | 8 | 8 | 15 | 9 | 10 | 5 | 11 | 11 | 9 | 9 | 0 | 1 | 1 | 1 | 0 | 0 | 0 | 0 |
