# Supplementary material for: Feeding Ecology in Oligocene Mylodontoid Sloths (Mammalia, Xenarthra) as Revealed by Orthodentine Microwear Analysis
Source: J Mamm Evol. 2017 Jul 28;25(4):551–64. doi: 10.1007/s10914-017-9405-x (PMC6209052; doi:10.1007/s10914-017-9405-x)
Supplement: Supplementary file 2 — (DOCX 19 kb) [file 10914_2017_9405_MOESM2_ESM.docx]

Feeding ecology in Oligocene mylodontoid sloths (Mammalia, Xenarthra) as revealed by orthodentine microwear analysis. Journal of Mammalian Evolution. Kalthoff DC*, Green JL

Department of Zoology, Swedish Museum of Natural History, Box 50007, SE–104 05 Stockholm, Sweden (DCK)

Department of Geology, Kent State University at Tuscarawas, 330 University Drive NE, New Philadelphia, OH 44663, USA (JLG)

* Corresponding author: Kalthoff DC, E-Mail: daniela.kalthoff@nrm.se

**Supplementary Table 2**

Raw stereoscopic microwear variables for all extant individuals. Two separate countings were executed per specimen, designated by either a 1 or 2 following the specimen number. Abbreviations: SP = number of small pits; LP = number of large pits; FS = number of fine scratches; CS = number of coarse scratches; G = gouges; PP = puncture pits; 0 = feature absent; 1 = feature present. Molariforms from the maxilla are denoted by MF, L = left, R = right. NRM = Swedish Museum of Natural History, Stockholm, Sweden.

| **Specimen** | **SP-1** | **SP-2** | **LP-1** | **LP-2** | **FS-1** | **FS-2** | **CS-1** | **CS-2** | **G-1** | **G-2** | **PP-1** | **PP-2** |
| --- | --- | --- | --- | --- | --- | --- | --- | --- | --- | --- | --- | --- |
| *Bradypus variegatus* |  |  |  |  |  |  |  |  |  |  |  |  |
| NRM 580502 | 10 | 11 | 22 | 14 | 7 | 13 | 7 | 8 | 1 | 1 | 0 | 0 |
| NRM 580538 | 14 | 13 | 12 | 11 | 9 | 11 | 10 | 9 | 1 | 1 | 0 | 0 |
| NRM 581032 | 8 | 11 | 10 | 11 | 1 | 10 | 2 | 10 | 1 | 0 | 1 | 0 |
| NRM 581211 | 7 | 12 | 12 | 6 | 11 | 2 | 9 | 6 | 1 | 0 | 0 | 0 |
| NRM 581503 | 8 | 18 | 10 | 10 | 3 | 8 | 10 | 10 | 0 | 0 | 0 | 0 |
| NRM 581552 | 9 | 11 | 7 | 12 | 6 | 10 | 4 | 6 | 0 | 0 | 0 | 0 |
| NRM 581557 | 9 | 11 | 9 | 10 | 11 | 6 | 4 | 10 | 0 | 1 | 0 | 0 |
| NRM 581564 | 13 | 12 | 12 | 6 | 9 | 6 | 9 | 1 | 1 | 0 | 0 | 0 |
| *Choloepus didactylus* |  |  |  |  |  |  |  |  |  |  |  |  |
| NRM 580717 | 6 | 9 | 6 | 8 | 6 | 7 | 5 | 7 | 1 | 1 | 0 | 0 |
| NRM 581540 | 12 | 8 | 5 | 6 | 7 | 5 | 7 | 9 | 1 | 1 | 1 | 1 |
| NRM 586554 | 7 | 8 | 6 | 5.5 | 6 | 6.5 | 3 | 8 | 1 | 1 | 1 | 1 |
| NRM 593602 | 6 | 8 | 7 | 5 | 8 | 6 | 8 | 12 | 1 | 1 | 0 | 0 |
| NRM 593606 | 8 | 10 | 7 | 4 | 10 | 14 | 6 | 3 | 0 | 0 | 0 | 0 |
| NRM 601111 | 8 | 9 | 5 | 4 | 7 | 8 | 4 | 2 | 1 | 1 | 0 | 0 |
