# Supplementary material for: Feeding Ecology in Oligocene Mylodontoid Sloths (Mammalia, Xenarthra) as Revealed by Orthodentine Microwear Analysis
Source: J Mamm Evol. 2017 Jul 28;25(4):551–64. doi: 10.1007/s10914-017-9405-x (PMC6209052; doi:10.1007/s10914-017-9405-x)
Supplement: Supplementary file 3 — (DOCX 19 kb) [file 10914_2017_9405_MOESM3_ESM.docx]

Feeding ecology in Oligocene mylodontoid sloths (Mammalia, Xenarthra) as revealed by orthodentine microwear analysis. Journal of Mammalian Evolution. Kalthoff DC*, Green JL

Department of Zoology, Swedish Museum of Natural History, Box 50007, SE–104 05 Stockholm, Sweden (DCK)

Department of Geology, Kent State University at Tuscarawas, 330 University Drive NE, New Philadelphia, OH 44663, USA (JLG)

* Corresponding author: Kalthoff DC, E-Mail: daniela.kalthoff@nrm.se

**Supplementary Table 3**

Raw microwear data for all SEM images. Two separate images were captured and counted per specimen, designated by either a 1 or 2 following the specimen number. Although MNHN-DES 250 includes three different teeth, only data from LMF4 was included in the text for comparison among different individuals of *Octodontotherium*. * = randomly selected images that were replicated in the random, blind image file and counted during the analysis to test intraobserver error. Molariforms from the maxilla are denoted by MF, L = left. MNHN = Musée National d’Histore Naturelle, Paris, France.

| **Specimen – Image#** | **FW** | **R** | **P** | **S** |
| --- | --- | --- | --- | --- |
| *Octodontotherium grande* |  |  |  |  |
| MNHN-DES 236 – 1 | 3.49 | 0.38 | 21 | 43 |
| MNHN-DES 236 – 2 | 2.19 | 0.215 | 34 | 49 |
| MNHN-DES 237 – 1 | 2.72 | 0.598 | 21 | 31 |
| MNHN-DES 237 – 2 | 2.17 | 0.766 | 25 | 51 |
| MNHN-DES 238 – 1 | 4.08 | 0.567 | 15 | 24 |
| MNHN-DES 238 – 2 | 2.89 | 0.47 | 18 | 55 |
| MNHN-DES 239 – 1 | 3.28 | 0.573 | 22 | 27 |
| MNHN-DES 239 – 1* | 3.05 | 0.43 | 24 | 30 |
| MNHN-DES 239 – 2 | 2.55 | 0.382 | 18 | 43 |
| MNHN-DES 245 – 1 | 2.23 | 0.727 | 14 | 55 |
| MNHN-DES 245 – 2 | 2.21 | 0.189 | 23 | 53 |
| MNHN-DES 245 – 2* | 2.42 | 0.375 | 20 | 53 |
| MNHN-DES 246 – 1 | 2.72 | 0.181 | 16 | 52 |
| MNHN-DES 246 – 2 | 2.99 | 0.289 | 16 | 55 |
| MNHN-DES 250 LMF2 – 1 | 2.76 | 0.624 | 16 | 41 |
| MNHN-DES 250 LMF2 – 2 | 2.27 | 0.527 | 26 | 49 |
| MNHN-DES 250 LMF2 – 2* | 2.46 | 0.642 | 31 | 41 |
| MNHN-DES 250 LMF3 – 1 | 2.09 | 0.446 | 18 | 48 |
| MNHN-DES 250 LMF3 – 2 | 2.89 | 0.837 | 24 | 42 |
| MNHN-DES 250 LMF4 – 1 | 3.41 | 0.263 | 22 | 33 |
| MNHN-DES 250 LMF4 – 1* | 3.21 | 0.276 | 20 | 33 |
| MNHN-DES 250 LMF4 – 2 | 2.26 | 0.269 | 15 | 46 |
| MNHN-DES 251 – 1 | 3.75 | 0.2 | 11 | 17 |
| MNHN-DES 251 – 1* | 3.29 | 0.146 | 15 | 20 |
| MNHN-DES 251 – 2 | 3.47 | 0.606 | 20 | 39 |
| MNHN-DES 251 – 2* | 3.89 | 0.508 | 24 | 36 |
| MNHN-DES 252 – 1 | 1.76 | 0.609 | 5 | 55 |
| MNHN-DES 252 – 1* | 1.42 | 0.632 | 7 | 55 |
| MNHN-DES 252 – 2 | 1.79 | 0.617 | 13 | 57 |
| MNHN-DES 252 – 2* | 1.61 | 0.647 | 15 | 65 |
| *Orophodon hapaloides* |  |  |  |  |
| MNHN-DES 260 – 1 | 1.13 | 0.223 | 3 | 72 |
| MNHN-DES 260 – 1* | 1.18 | 0.182 | 7 | 77 |
| MNHN-DES 260 – 2 | 1.19 | 0.077 | 7 | 67 |
| MNHN-DES 267 – 1 | 2.85 | 0.131 | 13 | 44 |
| MNHN-DES 267 – 2 | 1.74 | 0.481 | 14 | 64 |
| MNHN-DES 268 – 1 | 1.79 | 0.25 | 16 | 57 |
| MNHN-DES 268 – 2 | 4.76 | 0.175 | 23 | 38 |
| MNHN-DES 269 – 1 | 1.87 | 0.792 | 10 | 62 |
| MNHN-DES 269 – 2 | 2.93 | 0.719 | 22 | 57 |
| MNHN-DES 277 – 1 | 2.5 | 0.453 | 15 | 30 |
| MNHN-DES 277 – 2 | 2.83 | 0.539 | 12 | 39 |
| *Octodontotherium vel Orophodon* | |  |  |  |
| MNHN-DES 233 – 1 | 2.45 | 0.4 | 19 | 56 |
| MNHN-DES 233 – 2 | 1.94 | 0.532 | 12 | 49 |
| MNHN-DES 234 – 1 | 2.58 | 0.475 | 25 | 57 |
| MNHN-DES 234 – 1* | 2.68 | 0.49 | 25 | 58 |
| MNHN-DES 234 – 2 | 3.28 | 0.582 | 36 | 43 |
